# Nursing

**Published:** 1907-03

**Authors:** 


### NURSING.

Nursing has become a fine art. From a menial calling it has risen to the dignity of a learned profession; and to become an efficient and successful nurse one must acquire much medical and surgical knowledge, as well as a knowledge of general and special hygiene, and dietetics. The art, and I may say, science of nursing, is now taught *secundem artem,* and a diploma is granted upon successful completion of the prescribed course of study.

It is a long step from Sairy Gamp and Betsy Prigg (see Martin Chuzzlewitt) to the model trained nurse, graduated from the Chautauqua School of Nursing, Jamestown, N. Y., for instance; and even from Bill Nye's pretty hospital nurse, who skillfully "removed a porous plaster received during the war." She was in the mid-evolution stage, Sairy and Betsy were in the germinal, or larval, stage. Poor John Westbrook! While he raved in delirium, Sairy was snoozing in the easy chair,—tanked up on beer or whisky. It will be remembered, that she told Mr. Mould the undertaker, and Mrs. Mould, and the "Miss Moulds," "Whether I sicks or monthlys, ma'am, I *do* require it—which I makes free to confess, to be brought reg'lar and drawed mild." "You'll have to have his pillow," she said to her chum, Betsy Prigg, when she came to relieve her for the night.

The foregoing reflections have come from reading a book sent me by the Chautauqua School of Nursing. It consists of a series of twenty-six lectures each complete in itself, and so bound that any one lecture can be detached and replaced. It is the most complete course that can be imagined, and covers everything a nurse should know in order to be helpful to patient and doctor. Moreover, it is beautifully illustrated. The language is plain, clear and pleasing to read.

I do not know whether or not the book is for sale, and if so, the price of it. It is a gem, and it would be a blessing if every family could have a copy of it. I prize it very highly.

